# Genetic diversity in populations of African mahogany (*Khaya grandioliola* C. DC.) introduced in Brazil

**DOI:** 10.1590/1678-4685-GMB-2018-0162

**Published:** 2020-04-27

**Authors:** Sabrina Delgado Soares, Ludmila Ferreira Bandeira, Stela Barros Ribeiro, Mariana Pires de Campos Telles, João Augusto da Silva, Canrobert Tormin Borges, Alexandre Siqueira Guedes Coelho, Evandro Novaes

**Affiliations:** 1 Universidade Federal de Goiás, Instituto de Ciências Biológicas, Goiânia, GO, Brazil; 2 Universidade Federal de Goiás, Escola de Agronomia, Goiânia, GO, Brazil.; 3 Pontifícia Universidade Católica de Goiás, Escola de Ciências Agrárias e Biológicas, Goiânia, GO, Brazil.; 4 Mudas Nobres Company, Goiânia, GO, Brazil.

**Keywords:** Microsatellites, SSR, genetic structure, genetic divergence, hardwood

## Abstract

Given its high-valued wood, the African mahogany *(Khaya grandifoliola)* has been envisaged as a renewable source of tropical hardwoods in Brazil. However, there are concerns about the hypothesized low diversity among the few *K. grandifoliola* germplasm sources introduced in the country. Using eight microsatellite markers, we evaluated the genetic diversity and divergence among 53 superior trees selected from three provenances of *K. grandifoliola* located in the state of Para. These populations are among the oldest plantations (>15 years) in Brazil and, therefore, the country's main seed sources. The average number of alleles per locus was 5.9, expected heterozygosity was moderate (^=0.56) and lower than the high observed heterozygosity (H_O_=0.74). Therefore, the intrapopulation fixation index was negative (f=-0.31) indicating the possibility that selection of superior trees might have favored heterozygous plants with heterosis. No genetic structure was observed between provenances. The genetic diversity observed within selected trees, with an effective population size *(Ne)* of 30.4, is comparable to that of natural populations of African and Brazilian mahoganies. Therefore, our results contradict the idea that the genetic diversity of *K. grandifoliola* introduced in Brazil is low and show that our germplasm can be exploited for breeding purposes.

## Introduction

African mahoganies *(Khaya anthoteca, K. grandifolia, K. ivorensis, K. senegalensis),* from the Meliaceae family, are exotic prime wood species that can provide a renewable source of tropical hardwood. Although they are not as fast growing as *Eucalyptus* and *Pinus* species, African mahoganies exhibit excellent wood quality, with similar physicochemical properties to the Brazilian mahogany *(Swietenia macrophylla)* (Pinheiro *et al*., 2011). The genus *Khaya* is still in its wild state, having excellent potential for breeding and genetic gains (Nikles *et al*., 2008). Among the African mahoganies, *K. grandifoliola* C. DC. stands out for its faster growth, better natural pruning, and straighter stem (Pinheiro *et al*., 2011). In addition to these features, the species does not seem to be susceptible to the shoot borer *Hypsipyla grandella,* which precludes homogeneous plantations of the Brazilian mahogany (Falesi and Baena, 1999).

The various *K. grandifoliola* plantations established in the country originated from only five trees that were first introduced in 1976, at EMBRAPA Amazônia Oriental, and a few producers who imported seeds from different regions of Africa, where the species is native (Falesi and Baena, 1999). These trees were originally identified as *K. ivorensis*, but in 2015 a specimen from EMBRAPA was reclassified as *K. grandifoliola* by the Meliaceae botanists of the Kew Royal Botanic Gardens (Pennington and Cheek, 2015). Seed production from these initial plantations occurred in the 1990s, and interest on the species by Brazilian investors took off only after the year 2000. Given its recent introduction and the growing interest in African mahogany, new studies are needed in areas such as silviculture, wood technology, entomology, and forest pathology (Ribeiro *et al*., 2017).

Since there were few introductions of *K. grandifoliola,* there may be a founder effect in the Brazilian germplasm. In other words, the original diversity from African natural populations may not be well represented in our germplasm. Therefore, it is important to know the level of genetic diversity in the established plantations to assess their sustainability and the possibility of using this genetic resource in breeding programs. Given the few germplasms of the species introduced in Brazil, there are concerns about a possible low genetic diversity in the populations of *K. grandifoliola* established in the country (Ribeiro *et al*., 2017). Genetic diversity is important for long-term recurrent selection breeding programs, as was recognized by *P. radiata* breeders in New Zealand that re-introduced ~600 plus trees from the species’ entire geographic range (Burdon, 2008).

Despite its ecological importance, vulnerability of extinction in Africa, and its high wood value (ITTO, 2018), *K. grandifoliola* has been scientifically neglected. Few studies have been published for the species (Ribeiro *et al*., 2017), but none of them focused on the genetic diversity in natural populations of *K. grandifoliola.* The majority of the studies describe its medicinal potential, such as treatment of malaria (Agbedahunsi *et al*., 2004; Tepongning *et al*., 2011), isolation and structural characterization of limonoids (Zhang *et al*., 2009) that exhibit cytotoxic activity against tumor cells (Ji *et al*., 2014), antifungal and antimicrobial activity (Abdelgaleil *et al*., 2005), and as immunosuppressant (Zhang *et al*., 2012; Wang *et al*., 2014). More recently, given the growing interest in its wood, studies have analyzed its density, physical, and quality properties (Soranso *et al*., 2016; Mohd-Jamil *et al*., 2017; Vidaurre *et al*., 2017). To date, there is no genetic improvement study for *Khaya* species in Brazil. Worldwide, the only improvement program for the genus is with *K. senegalensis* in Australia (Nikles *et al*., 2008).

Studying the genetic variability between and within natural or cultivated populations is important for establishing strategies to conserve, domesticate and improve genetic resources (Chalmers *et al*., 1994; White *et al*., 1999; Lemes *et al*., 2011; Diniz-Filho *et al*., 2012). If a founder effect and low variability is, in fact, present among populations of *K. grandifoliola* introduced in Brazil, it is important to manage these populations to reduce risks of further genetic bottlenecks. Therefore, seed collections should be performed on an adequate number (> 20) of mother trees (Lengkeek *et al*., 2004). In addition, it is important to avoid seed collections in areas where selective logging of plus-trees was performed, since these selections may lead to possible dysgenic selection and genetic erosion of future plantations (Cornelius *et al*., 2005).

Molecular markers facilitate and improve the estimation of the degree of genetic variability, enabling significant advances in genetic studies of plant populations. Among the DNA markers, microsatellites (simple sequence repeats, SSRs) stand out because of their co-dominance, multiallelism, and high level of detectable polymorphism. As such, estimates of genetic differences can be obtained even between related individuals. The information obtained from these markers allows estimation of different genetic parameters, such as level of heterozygosity or diversity, as well as the degree of genetic structure and differentiation between different groups of individuals. This information is very useful in monitoring and managing the genetic variability available in natural ecosystems, germplasm banks, or genetic improvement programs (Grattapaglia, 2010).

The objective of the present study was to estimate the diversity and genetic structure of 53 selected trees from three provenances of *K. grandifoliola* that are, currently, the main seed sources for *K. grandifoliola* plantations in Brazil. Our hypotheses were: 1) the Brazilian germplasm of *K. grandifoliola* has low genetic diversity; 2) populations introduced from Africa (Ivory Coast and Tanzanian provenances) have higher genetic diversity than progenies of the first five *K. grandifoliola* trees introduced in the country; 3) populations introduced from different regions of Africa will present genetic structure. For the analyses of genetic diversity, we successfully transferred *K. senegalensis* microsatellites (Sexton *et al*., 2010; Karan *et al*., 2012) to *K. grandifoliola.*

## Materials and Methods

### Origin and sampling of biological material

A total of 53 *K. grandifoliola* trees were selected from three provenances located on two farms in the state of Pará, Brazil (Figure S1). The selection was made in February 2012, based on phenotypes directly related to wood yield: height and diameter at breast height (DBH). Mass selection was carefully performed, avoiding trees that benefitted from the lack of competition because of the death of neighbor trees.

Selection was performed in three provenances located in two farms with the oldest *K. grandifoliola* plantations in Brazil. The first two provenances were located at the farm belonging to Norton Amador Costa (1º28’45.69”S and 47º27’14.17”W). This farm contains more than 1500 trees (~13 years old in 2012) originating from two African provenances: Ivory Coast and Tanzania. The third provenance was located at the farm owned by Hiroshi Okajima (03º04’13.6”S and 47º28’58.9”W), whose trees (~19 years old in 2012) originated from seeds collected from the first five *K. grandifoliola* trees introduced in Brazil. These five trees were planted in 1976 at Embrapa Amazônia Oriental (Brazilian Agricultural Research Corporation, Eastern Amazon). Fiftythree superior quality trees were selected, 12 from the Mr Norton’s Ivory Coast provenance, 21 from Mr. Norton’s Tanzania, and 20 from Mr. Okajima’s. These trees were cloned and are being evaluated in clonal tests conducted in different regions of the country.

For this study, leaf samples were collected from selected trees. In the field, they were immediately packed in ice and then stored in a freezer (-80 ºC). Samples from a control group consisting of 12 *K. senegalensis* seedlings, obtained from a local nursery, were also used. These genotypes served as an external group in the analyses of genetic structure to compare with *K. grandifoliola* trees.

### DNA extraction and quantification

The Doyle and Doyle (1990) protocol was used for genomic DNA extraction. The quality of the extracted DNA was assessed in 1% agarose gel stained with ethidium bromide in a NanoDrop spectrophotometer (Thermo Scientific). The acceptable ratio between absorbances A260/A280 was 1.7 to 2.0 to avoid excessive contamination by protein and polysaccharide compounds. After quantification, the DNA samples were diluted (6 ng/µL) and reassessed in a NanoDrop spectrophotometer and 1% agarose gel.

### Selection and screening of SSRs

A literature search revealed the nonexistence of SSR primers developed for *K. grandifoliola.* However, for the congener *K. senegalensis,* two studies were found that describe 13 (Sexton *et al*., 2010) and 11 (Karan *et al*., 2012) sequences of SSR primer pairs. Given that high SSR transferability has been demonstrated between the genera *Swietenia* and *Khaya* (Lemes *et al*., 2011), we presumed that the primers developed for *K. senegalensis* would function efficiently in *K. grandifoliola.* These 24 primer pairs were synthesized and submitted to initial screening to assess their amplification in 3% agarose, with six DNA samples from six *K. grandifoliola* trees. Screening involved PCRs with a final volume of 13 µL, containing 9.0 ng of DNA, 1.5 pmol of each primer, 1 U *Taq* DNA polymerase, MgCl_2_ (3 mM), reaction buffer (1x), BSA (2.5 mg/mL), and dNTP (2.5 mM). PCR thermocycling included an initial denaturation at 95 ºC for 7 min, followed by 35 cycles with denaturation at 94 ºC for 30 s, annealing for 30 s at 50–55 ºC depending on the primer pair, and extension at 72 ºC for 1 min. After thermocycling, a final extension step was performed at 72 ºC for 5 min. Locus amplification was conducted in a T100 Thermal Cycler (Bio-Rad).

PCR products visualized in 3% agarose gel were obtained for 21 of the 24 SSR loci assessed in this screening. For 19 of the 21 SSR loci, primers were labeled with either 6-FAM, HEX or NED-Replacement fluorescent dyes (Alpha DNA, Canada) for the genotyping of this study.

### Amplification of SSR markers

The 19 pairs of SSR primers labeled with fluorescent dye were submitted to further screening via capillary electrophoresis, using the DNA of six individuals. Based on this screening, eight easily genotyped polymorphic loci (Table 1) were selected for PCR amplification in the full study population. PCR was conducted in a multiplex system (duplex) using the PCR Multiplex Kit (Qiagen), as described in the kit manual. The PCR program used to screen the loci was performed as described in the previous section for the SSR screening.

**Table 1:** Primers used to amplify SSR markers in *K. grandifoliola*, with their respective fluorochromes, annealing temperature (Ta, ºC) and multiplex.

Primer*	Fluorochrome	Ta	Multiplex
Ks022	6-FAM	55ºC	Duplex 1
Ks051	HEX	55ºC	Duplex 1
Ks086	NEDr	55ºC	Duplex 2
ssrKs09	6-FAM	55ºC	Duplex 2
ssrKs15	6-FAM	55ºC	Duplex 3
ssrKs7	HEX	55ºC	Duplex 3
Ks008	NEDr	50ºC	Duplex 4
Ks040	6-FAM	50ºC	Duplex 4
ssrKs16	6-FAM	50ºC	Isolate

Marker identifications initiating with Ks were developed by Sexton *et al*. (2010) and those with ssrKs were from Karan *et al*. (2012).

Capillary electrophoresis was performed in an ABI-3100 genetic analyzer (Applied Biosystems). The electropherograms were analyzed for genotyping in the GeneMapper 3.5 program (Applied Biosystems). The genotypes were organized in a spreadsheet, with microsatellite loci on the lines and individuals (selected trees) in the columns.

### Population genetics analyses

For population genetics and statistical analyses, the Norton population was subdivided into two groups: one with 12 trees from the Ivory Coast and another with 21 trees from Tanzania. Thus, analyses were carried out considering the Okajima, Norton Tanzania (Tz), and Norton Ivory Coast (IC) provenances as subpopulations, totaling 53 selected individuals.

Analyses of genetic diversity were conducted using the Genetic Data Analysis 1.0 (GDA) (https://phylogeny.uconn.edu/software/) and FSTAT 2.9.3.2 programs (https://www2.unil.ch/popgen/softwares/fstat.htm). The GDA program estimates the number of alleles per polymorphic loci *(Ap),* observed (H_O_) and expected (H_E_) heterozygosity, and the intrapopulation fixation index (*f*). The FSTAT program was used to estimate the allelic richness (*Ar*) of the subpopulations. Effective population size was estimated with NeEstimator v2 (Do *et al*., 2014), using the linkage disequilibrium method (Waples and Do, 2010), with random mating and a critical allele frequency of 2%.

Since the markers were originally developed for *K. senegalensis* (Sexton *et al*., 2010; Karan *et al*., 2012), their usefulness in *K. grandifoliola* was evaluated by estimating their probability of genetic identity *(PI)* (Paetkau and Strobeck, 1994) and of paternity exclusion *(PE)* (Weir, 1996) in the studied populations. A useful batch of markers should provide very low values of PI (the probability of two unrelated individuals presenting the same genotype) and very high values for PE (the probability that a non-genitor be detected as such in a paternity test). These estimates were obtained with Identity 4.0 software (https://homepage.uni-graz.at/de/kristina.sefc/).

The genetic structure among subpopulations (provenances) was assessed with the GDA program using the Weir and Cockerham (1984) estimators of the Wright (1965) Fstatistics. The estimated parameters were *F,* theta (θ) and *f,* corresponding to Wright’s total fixation (F_IT_), interpopulation (F_st_), and intrapopulation *(F_IS_)* fixation indexes, respectively.

The possibility of genetic structure among samples was further investigated with a principal component analysis (PCA) to depict the genetic distance among samples. The PCA was performed in R with package ape (Paradis *et al*., 2004). The genetic distance between selected trees was calculated using the method of Rogers (1972) modified by Wright (1978). A Mantel test was performed to evaluate the correlation between the cophenetic and genetic distance matrices.

The genetic structure was also assessed by the Structure 2.3.4 program, which uses a Bayesian approach (Pritchard *et al*., 2000). The Structure analysis was performed with a burn-in of 50,000 and 500,000 randomizations applying the Markov chain Monte Carlo (MCMC) method. We applied a model that predicts the possibility of admixture between the subpopulations and a model of correlated allelic frequencies. Simulations were performed using *K* (number of sub-populations or clusters) varying from one (no genetic structure) to eight, with 30 iterations for each K. The best *K* value was assessed using the method developed by Evanno *et al*. (2005) using the Structure Harvester program (Earl and von Holdt, 2012).

## Results

Of the 19 *K. senegalensis* SSR markers synthesized with fluorescent dyes for the study, only nine exhibited a good allelic amplification pattern in capillary electrophoresis (Table 1). Of these, four duplex systems were established and the ssrKs16 marker was analyzed separately. During genotyping in the GeneMapper program, the Ks008 marker of duplex 4 (Ks040 and Ks008) showed a dubious and inconsistent allelic pattern. Hence, we excluded this marker from further analyses.

For the eight remaining loci, 47 alleles were found in the population of 53 selected trees. The average number of alleles per locus (*A_P_*) was 5.9, ranging between 3 (locus ssrKs16) and 16 alleles (locus Ks040). Average heterozygosity was 0.738, with locus ssrKs7 exhibiting the highest observed heterozygosity (*Ho*= 0.979) and Ks022 the lowest (*H_o_*= 0.442). Expected heterozygosity *(H_e_)* was lower than observed (*H_O_*), with a mean of 0.563, varying from 0.365 for locus Ks22 to 0.835 for Ks040. Given the greater heterozygote frequency than expected by Hardy-Weinberg equilibrium, the estimated intrapopulation fixation index (*f*) was negative and equal to -0.314 (Table 2).

**Table 2:** Estimates of genetic diversity parameters *(Ap, He, Ho* and *f)* and probabilities *(PE* and *PI)* for the eight SSR markers genotyped in the 53 *K. grandifoliola* selected trees.

SSR	*N*	*A_P_*	*He*	*Ho*	*f*	*PE*	*PI*
Ks022	52	5	0.365	0.442	-0.211	0.180	0.444
ssrKs7	48	7	0.811	0.979	-0.209	0.605	0.068
Ks86	51	4	0.498	0.666	-0.342	0.214	0.352
ssrKs15	52	4	0.523	0.923	-0.777	0.211	0.347
ssrKs09	52	4	0.457	0.500	-0.092	0.243	0.339
Ks51	51	4	0.503	0.588	-0.169	0.238	0.328
ssrKs16	53	3	0.514	0.924	-0.812	0.199	0.361
Ks040	44	16	0.835	0.886	-0.061	0.663	0.050
Average	50.375	5.875	0.563	0.738	-0.314	0.970	7.612 x 10^-6^

*N =* number of individuals successfully genotyped with each marker; *Ap* = number of observed alleles; *He* = expected heterozygosity; *Ho* = observed heterozygosity; *f*= intrapopulation fixation index. *PE* = probability of paternity exclusion; *PI* = probability of identity.

Since the SSR markers were originally developed for *K. senegalensis* (Sexton *et al*., 2010; Karan *et al*., 2012), we assessed their transferability to *K. grandifoliola* by estimating the probability of paternity exclusion (*PE*) and identity (*PI*). The value of *PE* varied from 0.180 (Ks022) to 0.663 (Ks040) for individual loci and was 0.970 considering all the loci. *PI*, considering all the loci, was low (7.612 x 10^-6^), indicating that these markers are also useful for genotyping *K. grandifoliola*.

Genetic diversity analyses for the population of Norton trees subdivided into two provenances showed little difference between them (Table 3). Norton_ IC obtained *H_o_* = 0.746, and the Norton_Tz subpopulation 0.710, close to the values found in the Okajima provenance (0.758).

**Table 3:** Genetic diversity estimates (Ap, *He, Ho* and Ar) and intrapopulation fixation index (*f*) based on eight SSR markers for Okajima, Norton Tanzania and Norton Ivory Coast provenances.

Subpopulation	*N*	*A_P_*	*He*	*Ho*	*f*	*Ar*
Okajima	19.5	4.62	0.586	0.758	-0.304	3.946
Norton Tz	19.0	4.37	0.553	0.710	-0.296	3.896
Norton IC	11.9	3.00	0.550	0.746	-0.377	2.958
Average	16.8	4.00	0.563	0.738	-0.324	3.600

*N* = average number of individuals genotyped per locus; *AP* = average number of alleles per polymorphic locus; *He* = expected heterozygosity (Nei’s genetic diversity); *Ho* = observed heterozygosity; *f* = intrapopulation fixation index; *Ar* = average allelic richness considering 11 individuals.

Average *H_E_* also varied little between the Norton_IC *(H_E_* = 0.550) and Norton_Tz *(H_E_* = 0.553) provenances. These values are also close to that of the Okajima subpopulation, which exhibited *H_E_* = 0.586. Estimated allelic richness (*Ar*), calculated based on 11 individuals per subpopulation, was greater for the Okajima (*Ar* = 3.946) and Norton_Tz (*Ar* = 3.896) subpopulations compared to the Norton_IC (*Ar* = 2.958) subpopulation. These results contradict the hypothesis of low variability between *K. grandifoliola* populations introduced in Brazil.

Estimate of linkage disequilibrium-based contemporary effective population size *(Ne)* was 30.4 for all 53 samples, with 95% confidence interval ranging from 18.3 to 58.2. Considering each subpopulation individually, the Okajima population had *Ne* of 11.5, Norton_Tz of 14.2 and Norton_IC of 22.8. However, the confidence interval for the estimates of *Ne* for each subpopulation were high, making these estimates unreliable.

The estimate of interpopulation fixation index (θ = 0.008) was not statistically significant, with a 95% confidence interval varying from -0.007 to 0.028. This result indicates little differentiation or genetic structure between the subpopulations. The average intrapopulation fixation index (*f*) ranged from -0.377 (Norton_IC subpopulation) to -0.296 (Norton_Tz subpopulation), with an average of -0.324. The total fixation estimate *(F)* was also high and negative, with an average of -0.311, influenced by the high negative *f* value.

**Figure 1:**
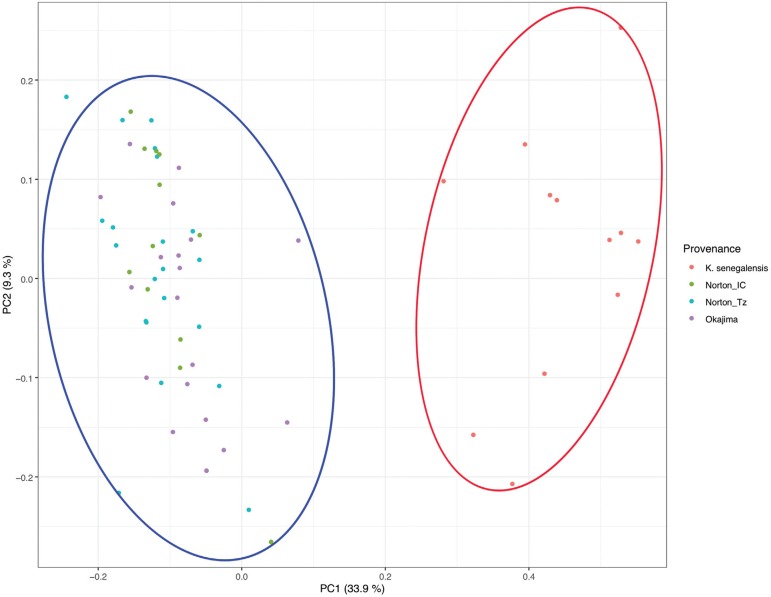
Genetic distance between samples obtained from a principal component analyses (PCA), with the first component (PC1) in the x-axis and the second (PC2) in the y-axis. The biplot depicts a strong genetic structure between species but not between the *K. grandifoliola* provenances. PCA was performed with a genetic distance matrix using Rogers (1972) modified by Wright (1978). Cophenetic correlation was high (r = 0,90) and significant (p-value < 0.0001) by the Mantel test.

Another indication of low genetic structure was observed from a principal component analyses (PCA) of the Roger’s genetic distance between the samples (Figure 1). The PCA biplot is a good representation of the genetic distances, as the cophenetic correlation was high (r = 0,90) and significant (p-value < 0.0001) according to the Mantel test. From the PCA biplot it becomes clear that there are only two clusters of genotypes, separating the two species (K. *grandifoliola* and *K. senegalensis).* Therefore, there is no apparent sub-population clusters among *K. grandifoliola* individuals. As such, no apparent genetic difference was observed between the three provenances (Okajima, Norton_IC and Norton_Tz) (Figure 1).

The results obtained from the Structure program also suggested the nonexistence of genetic structure between the 53 *K. grandifoliola* selected trees. The Evanno *et al*. (2005) method was used to determine the number of subpopulations (K), showing a higher ΔK for *K* = 5. However, the highest value obtained was very low (ΔK < 8) (Figure S2). Under the hypothesis that K=5, analysis of the bar graph (Figure S2) with the allocation of 53 individuals in the five populations, indicated that all the individuals had uniform distributions of allocation probability. This result provides, therefore, strong evidence for the absence of structure between the genotypes. A more detailed analysis of the estimated average and standard deviation of the likelihood of the different models (*K* ranging from 1 to 8) indicates that the most likely model is the one with *K* = 1 (absence of structure). This model exhibited the highest likelihood and lowest standard deviation among the 30 repetitions assessed (Figure S2). It is important to underscore that the Evanno *et al*. (2005) ΔK method is unable to assess absence of genetic structure.

## Discussion

Our results contradict the hypothesis of low genetic diversity in the *K. grandifoliola* populations introduced in Brazil. The population of 53 *K. grandifoliola* individuals exhibits relatively high genetic diversity *(H_E_* = 0.563 and *H_O_* = 0.738). Curiously, the 20 genotypes selected from the Okajima provenance, which originated from only five parental trees, had the highest levels of genetic diversity *(H_E_* = 0.586 and *H_O_* = 0.758). This result indicates that the five parental trees that originated the Okajima subpopulation were likely genetically diverse.

This finding is significant because it should alleviate concerns among producers of a possible low genetic diversity that might compromise the sustainability of African mahogany plantations *(K. grandifoliola)* in Brazil. It is important to underscore that the populations of the present study (Okajima and Norton) are among the only ones in the country at reproductive age. As such, these farms are the major sources of seeds and account for nearly all the *K. grandifoliola* seedlings currently produced in Brazil. This relatively high genetic diversity can be exploited for breeding purposes. In addition, it justifies plantations with higher density (3 x 2 m, for example) to allow selection of the best trees during thinning. The stems of many *K. grandifoliola* genotypes have tortuosity problems (Carmo *et al*., 2018). Thinning these trees should improve the sawn wood productivity. Nevertheless, the cost with seedlings and fertilization should increase at higher densities.

High diversity can be observed, primarily when the results of this investigation are compared with studies of genetic diversity in natural populations of economically important tropical wood species. Studies on phylogenetically close tree species, belonging to the family *Meliaceae,* showed similar *H_O_* and *H_E_* values to those obtained for *K. grandifoliola.* The genotyping of 10 microsatellite loci in a sample of 121 *Swietenia macrophylla* (Brazilian mahogany) adult trees from the Amazon forest found average *Ho* and *H_E_* values of 0.73 and 0.84, respectively (Lemes *et al*., 2002). Another study that sampled seven natural populations of *S. macrophylla* in the southern Amazon basin obtained *Ho* and *H_E_* values of 0.75 and 0.85 (Lemes *et al*., 2003). A third study conducted with 100 *S. macrophylla* trees sampled in Costa Rica forests and using five microsatellite loci obtained average *HO* and *H_E_* of 0.508 and 0.518 (Céspedes *et al*., 2003). By sampling 192 *Cabralea canjerana* (Meliaceae) trees in seven fragments of the Atlantic Forest in the state of Minas Gerais, Melo and Franceschinelli (2016) observed *H_O_* and *H_E_* of 0.70 and 0.73, using six microsatellite loci.

It is important to highlight that these studies were conducted with natural populations of Brazilian mahogany (*S. macrophylla*) and that the diversity values observed are close to those obtained in the selected trees of *K. grandifoliola* from this study *(H_O_* = 0.738 and *H_E_* = 0.563). This demonstrates that, although the selected trees originate from provenances with a restricted genetic base, their genetic diversity is comparable to that observed in natural populations of species belonging to the family *Meliaceae*.

To date, no genetic studies have been carried out in populations of *K. grandifoliola.* Research on *K. senegalensis* showed heterozygosity values similar to those obtained here. Lemes *et al*. (2011), using ten microsatellite loci to study 237 trees from 12 natural subpopulations of *K. senegalensis* from Benin, obtained average *H_O_* and *H_E_* of 0.486 and 0.484, respectively. Karan *et al*. (2012) used 12 microsatellites to analyze 73 *K. senegalensis* accessions from 11 countries encompassing the natural distribution of the species. The average *H_O_* and *H_E_* obtained were 0.621 and 0.739. In a more recent study, samples of 503 *K. senegalensis* individuals were collected from an area covering the entire natural distribution of the species in Africa. The genotyping of 13 microsatellite markers produced average values of 0.631 and 0.639 for *H_O_* and *H_e_,* respectively (Sexton *et al*., 2015). These results confirm that the 53 *K. grandifoliola* trees selected in our study exhibit comparable or only slightly lower genetic diversity *(H_O_* = 0.738 and *H_E_* = 0.563) than that obtained in natural populations of phylogenetically close species.

The estimate of heterozygosity, together with the number of alleles per locus and allelic richness, indicate that all three provenances (Okajima, Norton_IC and Norton_Tz) display genetic diversity with similar magnitudes. The magnitude of diversity demonstrates that these populations can be exploited for breeding purposes.

In this study, the average number of alleles per microsatellite locus observed in the population of *K. grandifoliola* (5.9 alleles per locus) was lower than estimates reported in research involving *K. senegalensis,* which ranged from 8.5 to 10.8 alleles per locus (Sexton *et al*., 2010; Lemes *et al*., 2011; Karan *et al*., 2012). These studies used a larger number of individuals than ours. Lemes *et al*. (2003) analyzed eight microsatellites in 194 *S. macrophylla* trees and found 147 genotyped alleles, with an average of 18.4 alleles per locus. On the other hand, Sexton *et al*. (2015) sampled the natural distribution of *K. senegalensis* in Africa (N = 503) and found 6.6 alleles per locus. A larger number of alleles results in greater capacity to generate new genotype combinations, thereby broadening the genetic base for both conservation and breeding purposes (Kageyama *et al*., 2003).

Therefore, although the selected population of *K. grandifoliola* exhibits moderate to high heterozygosity, the number of alleles per locus is lower than what have been found in studies involving other mahogany species. This lower allelic diversity suggests the need to reintroduce greater genetic variability from the origin of the species in Africa. New introductions of *K. grandifoliola* from African populations should broaden the genetic diversity of our germplasm, reducing its founder effect. Increased genetic diversity in *K. grandifoliola* is important to guarantee the sustainability and adaptability of plantations in different parts of the country. In addition, for genetic improvement programs, diversity enables long-term selection gains. However, the estimated effective population size of 30.4 is sufficient to guarantee many generations of genetic gains (Souza Jr *et al*., 2000) with recurrent selection in the populations already available in Brazil.

This said, it is important to recognize that there may be a founder effect in our germplasm. However, since there is no genetic study currently performed with natural populations of *K. grandifoliola,* we cannot test this hypothesis. Nonetheless, because of the few introductions, with few specimens of *K. grandifoliola*, a founder effect is likely. As such, new introductions from its natural range should increase the genetic diversity available in Brazil. In addition, it is important that seeds are collected from at least 20 mother trees for seedling production (Lengkeek *et al*., 2004), in order to avoid future inbreeding and genetic erosion in our populations of *K. grandifoliola.* Seed collection on areas where selective logging of plus-trees was performed should also be avoided, in order to prevent possible dysgenic selection (Cornelius *et al*., 2005).

When the Okajima, Norton_IC and Norton_Tz provenances were compared, they exhibited approximately the same values for *H_O_* and *H_E_.* The number of alleles per locus and allelic richness showed slightly discrepant values, with the Norton subpopulation from Ivory Coast (IC) obtaining the lowest number of alleles. The Norton_IC obtained lower *Ap* (3.000) and *Ar* (2.958) values than those observed in the Okajima subpopulation (Ap = 4.625 and *Ar* = 3.946). On the other hand, the Norton_Tz population had values closer (Ap = 4.375 and *Ar* = 3.896) to those obtained for the Okajima population.

These results were surprising since they contradict the initial hypothesis that the provenances collected from Africa (Norton Ivory Coast and Tanzânia) would display greater diversity than that of the Okajima. The Okajima provenance originated from only five parental trees located at Embrapa Amazônia Oriental. These five trees were the first *K. grandifoliola* introduced in the country and there are no reports of adult populations, in the proximity, that could pollinate these parental trees.

The intrapopulation fixation index is an important measure in population genetics studies, since it may indicate the presence of endogamy and other forces that cause deviations in the expected equilibrium between homozygotes and heterozygotes (Kageyama *et al*., 2003). In all the studied provenances, the estimated index was significantly negative, varying from -0.304 for Okajima to -0.377 for Norton_IC. The negative value of *f* indicates that heterozygote frequency is higher than expected by the Hardy-Weinberg equilibrium. Inbreeding depression is especially prevalent in adult populations of perennial species (Miller and Gross, 2011), and may decrease homozygote frequency, which, in turn, make the *f* value negative. Moreover, the fact that the trees in this study were selected for their greater growth may have favored more heterozygotic individuals with possible hybrid vigor. This is supported by the higher observed heterozygosity compared to what was expected by chance (i.e., under Hardy-Weinberg Equilibrium).

Analysis of 73 accesses from natural populations of *K. senegalensis* (Karan *et al*., 2012) also found a lower-thanexpected *H_O_* value (*f* = -0.160). Negative values, closer to zero (*f* = -0.038), were also reported in another investigation with *K. senegalensis* (Lemes *et al*., 2003). On the other hand, Sexton *et al*. (2015), also studying the African mahogany *K. senegalenis,* obtained a near zero, positive value of*f* (0.015). Similar findings were recorded in several natural populations of *S. macrophylla,* with average *f* values of 0.015 (Cespedes *et al*., 2003), 0.038 (Lemes *et al*., 2003), 0.024 (Lowe *et al*., 2003) and 0.149 (Novick *et al*., 2003).

Our results also indicate a low genetic structure between the *K. grandifoliola* provenances. The genetic diversity present in the selected population of *K. grandifoliola* is largely concentrated within (and not between) provenances. The partition of genetic variability between and within populations (Weir and Cockerham, 1984) showed no significant differences between subpopulations. Only 0.8% (0 = 0.008) of variability is attributed to differences between provenances and this value does not differ statistically from zero. This small genetic differentiation is consistent with the natural populations of African mahogany. A recent study with 503 *K. senegalensis* trees distributed in 19 areas of Africa, obtained an *F_st_* value of 0.013, also indicating low genetic differentiation between populations (Sexton *et al*., 2015).

The low genetic differentiation between provenances of *K. grandifoliola* is also corroborated by the results of the principal component analyses from the genetic distance matrix (Figure 1), as well as by the Bayesian approach implemented in the Structure software. This result is important because the Structure analysis is performed independent of the information of pre-established subpopulations. Therefore, Structure could have identified any other source of genetic structure different from the provenances where the samples were collected. However, this was not the case, and no significant genetic structure was observed among *K. grandifoliola*.

This low genetic structure among the 53 *K. grandifoliola* trees contradicts the initial hypothesis that provenances could exhibit genetic structure since they were from different origins. As such, the Okajima subpopulation is not significantly different from the provenances of Norton Ivory Coast (IC) and Tanzania (Tz), both in terms of diversity level and genetic differentiation. Thus, it is highly likely that the seeds used to plant the first five trees at Embrapa Amazônia Oriental, which supplied Mr. Okajima’s farm, and the seeds bought in Africa that supplied the Norton farm, came from the same population of *K. grandifoliola,* or from genetically connected populations in Africa. Another important factor to underscore is that Tanzania, a country from which the Norton_Tz seeds originated, does not contain natural populations of *K. grandifoliola*. Thus, this Tanzania subpopulation must have originated from the same population that gave rise to the other provenances.

To the best of our knowledge this is the first report of a genetic study in *K. grandifoliola*. The information obtained in this study regarding the breeding population of *K. grandifoliola* is important because it shows that the genetic diversity is not low, contradicting initial worries of a possible lack of genetic variation that could compromise the long-term sustainability of plantations in Brazil. We also demonstrated that the introduced provenances of *K. grandifoliola* do not seem to have genetic structure. This information is important for the conservation of genetic resources of the species in the country, as well as to guide future genetic improvement in *K. grandifoliola*. Knowing the genetic variability between and within populations is essential to strengthen genetic improvement and germplasm conservation programs (Diniz-Filho *et al*., 2012).

## Conclusions

The following conclusions could be drawn. First, microssatellite primers designed for *K. senegalensis* were successfully transferred to *K. grandifoliola.* Second, the level of genetic diversity between *K. grandifoliola* trees from the Okajima and Norton provenances, which are the primary sources of seeds in Brazil, can be considered at least moderate, contradicting the initial hypothesis of low diversity in the country’s plantations. Third, this genetic diversity is comparable to that observed in natural populations of African and Brazilian mahoganies, enabling exploitation of these *K. grandifoliola* selected trees in a breeding program. And fourth, there is no genetic structure among selected trees, indicating that the different *K. grandifoliola* introductions in Brazil may all have originated from the same African sources.

## Supplementary material

The following online material is available for this article:

Figure S1 – Localization of the two farms (Norton’s and Okajima’s), in Para state (Brazil), where the 53 trees were selected.

Figure S2 – Results from Structure depicting the likelihood for the different number of clusters (k).
