# Supplementary material for: Genetic diversity in populations of African mahogany (Khaya grandioliola C. DC.) introduced in Brazil
Source: Genet Mol Biol. 2020 Apr 27;43(2):e20180162. doi: 10.1590/1678-4685-GMB-2018-0162 (PMC7198008; doi:10.1590/1678-4685-GMB-2018-0162)
Supplement: Figure S1 [file 1415-4757-gmb-43-2-e20180162-suppl01.pdf]

Supplementary material to" Genetic diversity in populations of African mahogany (*Khaya grandioliola* C. DC.) introduced in Brazil"

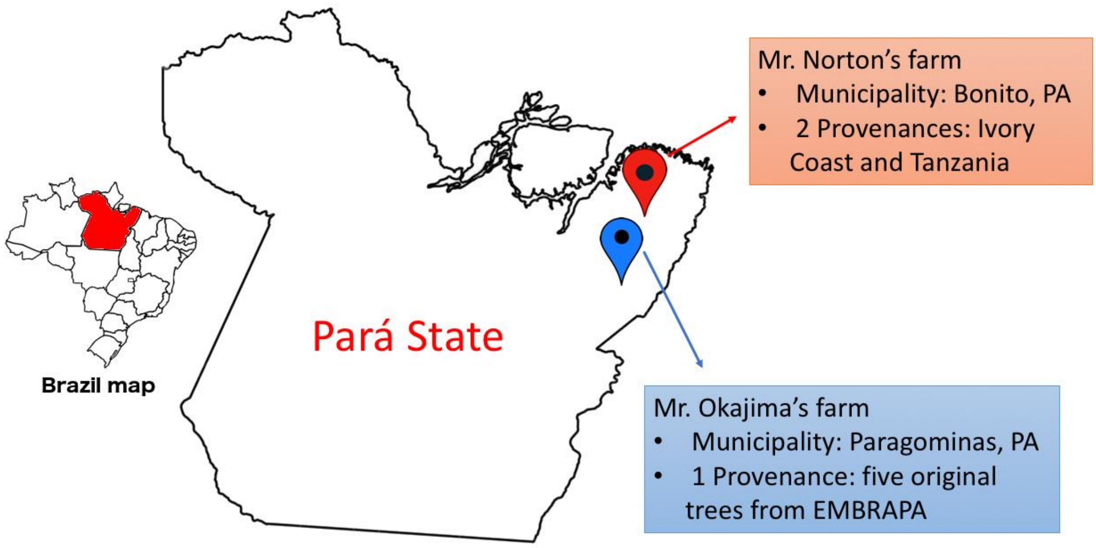

Figure S1 – Localization of the two farms (Norton's and Okajima's), in Pará state (Brazil), where the 53 trees were selected. Norton's trees were from two provenances: Ivory Coast and Tanzania. Okajima's trees were from the first five K. grandifoliola trees introduced in Brazil, planted in Embrapa Amazônia Oriental (Belém, PA, Brazil). The two farms are approximately 180 km apart.
