# Supplementary material for: Genetic diversity in populations of African mahogany (Khaya grandioliola C. DC.) introduced in Brazil
Source: Genet Mol Biol. 2020 Apr 27;43(2):e20180162. doi: 10.1590/1678-4685-GMB-2018-0162 (PMC7198008; doi:10.1590/1678-4685-GMB-2018-0162)
Supplement: Figure S2 [file 1415-4757-gmb-43-2-e20180162-suppl02.pdf]

Supplementary material to" Genetic diversity in populations of African mahogany (*Khaya grandioliola* C. DC.) introduced in Brazil"

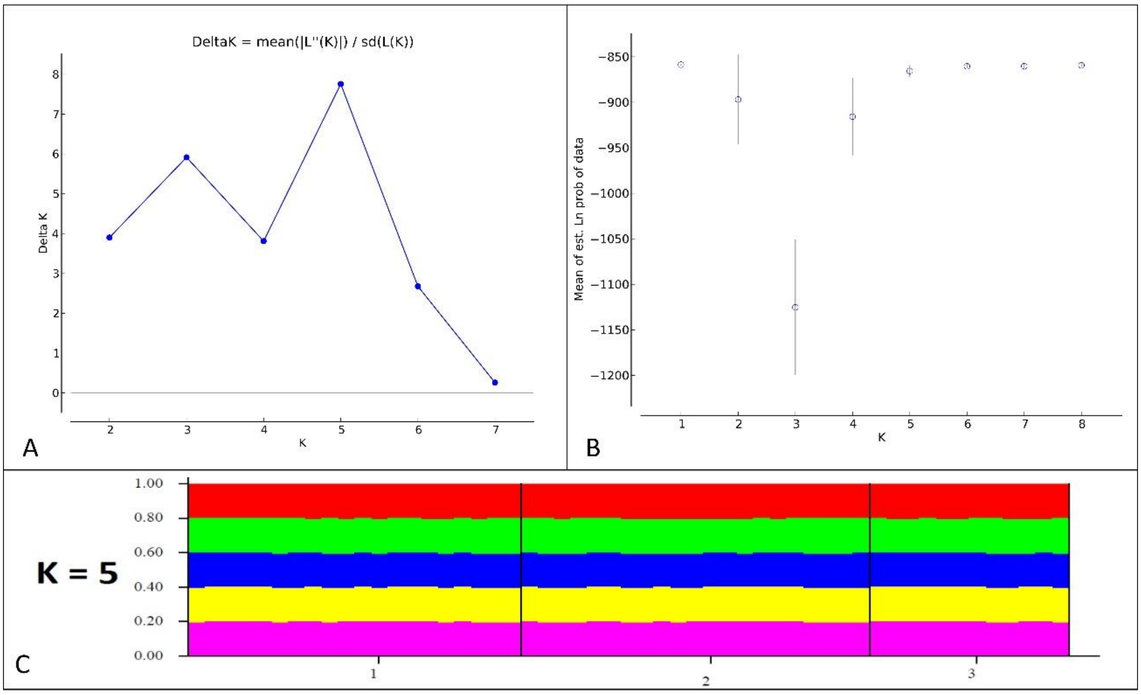

Figure S2 – Results from Structure depicting the likelihood for the different number of clusters (k). Values of *ΔK* for *K* ranging from 2 to 7, calculated according to Evanno *et al*. (2005) and the Structure bar graph. A: highest *ΔK* value observed for *K* = 5 among the 53 *K. grandifoliola* individuals selected. B: mean and standard deviation of the likelihoods of the different models obtained with *K* varying from 1 to 8, indicating K =1 as the most likely model. C: Structure bar graph, for K = 5, indicating absence of genetic structure among the individuals of the three *K. grandifoliola* provenances analyzed: 1 – Okajima, 2 – Norton_Tz and 3 – Norton_IC.
